# Behavior of mitral regurgitation after isolated tricuspid valve surgery: determinants and hemodynamic correlates

**DOI:** 10.1186/s13019-026-04302-7

**Published:** 2026-06-04

**Authors:** M. Savari, S. S. Eftekhari, S. Hosseini, H. Bakhshandeh, H. Khesali, R. Kaviani

**Affiliations:** 1 Cardiovascular Research Centre, Rajaie Cardiovascular Institute, Tehran, Iran; 2 Department of Cardiology, School of Medicine, Iran University of Medical Sciences, Tehran, Iran; 3 Cardiovascular Research Center, Hashemi Rafsanjani Hospital, Tehran, Iran; 4 Heart Valve Disease Research Center, Rajaie Cardiovascular Institute, Tehran, Iran; 5 Cardiovascular Epidemiology Research Center, Rajaie Cardiovascular Institute, Tehran, Iran; 6 Echocardiography Research Center, Rajaie Cardiovascular Institute, Tehran, Iran; 7 Cardiovascular Imaging Research Center, Rajaie Cardiovascular Institute, Tehran, Iran

**Keywords:** Tricuspid Valve Insufficiency, Mitral Valve Insufficiency, Echocardiography, Hemodynamics

## Abstract

**Background:**

Isolated tricuspid regurgitation (TR) is increasingly recognized as a clinically important disease associated with adverse outcomes. Surgical repair often improves right-sided hemodynamics, but its effects on mitral regurgitation (MR) are unclear. Early observations suggest that MR may worsen postoperatively, yet reliable predictors remain insufficiently defined.

**Objectives:**

To evaluate postoperative MR behavior following isolated surgical TR repair and identify echocardiographic predictors of MR worsening.

**Methods:**

This retrospective cohort study included patients ≥ 14 years who underwent isolated tricuspid valve repair between 2012 and 2023 at a tertiary center. Complete paired pre- and postoperative echocardiograms were analyzed by a blinded cardiologist. MR worsening was defined as an increase of ≥ 1 grade. Paired nonparametric tests compared pre- and postoperative echocardiographic indices, and between-group analyses examined factors associated with MR progression.

**Results:**

Nineteen patients (mean age 36.3 years; 63% male) met the inclusion criteria. TR severity improved significantly after surgery (*p* < 0.001), accompanied by marked right-ventricular reverse remodeling, including significant reductions in RV size (*p* = 0.001) and RVIDd (4.60 ± 0.58 to 3.89 ± 0.44 cm; *p* < 0.001). MR severity changed significantly in paired analysis (*p* = 0.010), and 63% of patients demonstrated postoperative worsening. Left-sided chamber dimensions and systolic function remained stable. Among all parameters assessed, only postoperative E/e′ differed significantly between patients with and without MR worsening (9.39 ± 2.32 vs. 7.16 ± 0.65; *p* = 0.045).

**Conclusions:**

In this small exploratory cohort, postoperative MR worsening occurred in a notable subset of patients, although the clinical relevance of these changes remains uncertain.Among the evaluated parameters, postoperative E/e′ was higher in patients with MR worsening, suggesting a possible association between elevated LV filling pressures and postoperative MR behavior. Routine postoperative surveillance may be warranted to detect clinically relevant MR changes.

## Introduction

Tricuspid regurgitation (TR) has historically been referred to as the forgotten valve. However, accumulating evidence demonstrates that severe TR is an independent predictor of mortality, even in the absence of left-sided heart disease [1–4]. Although TR frequently coexists with mitral or aortic valve disease, isolated TR (iTR) is increasingly recognized as a distinct clinical entity associated with substantial morbidity [5, 6]. Despite advances in surgical techniques, isolated tricuspid valve surgery (TVSx) remains associated with higher perioperative and mid-term complication rates compared with left-sided valve procedures [2, 6–8]. Consequently, current guideline recommendations for iTR rely largely on expert consensus, reflecting the limited availability of high-quality evidence to guide timing and indications.

Physiologically, chronic iTR, and to a lesser extent severe tricuspid stenosis (TS), leads to reduce left ventricular (LV) preload, which may mask or underestimate the true severity of mitral regurgitation (MR) [9]. After isolated tricuspid valve repair, improved RV forward flow and restoration of LV preload may unmask previously underestimated MR or precipitate its progression, a phenomenon described in several clinical series [9]. Although concomitant tricuspid repair is recommended during left-sided valve surgery when annular dilation is present [10, 11], there is no consensus regarding whether mild-to-moderate MR should be addressed at the time of isolated tricuspid valve surgery [5, 10]. Only limited retrospective studies, primarily from East Asia, have evaluated the interaction between TR correction and subsequent MR evolution, often relying on advanced echocardiographic strain analyses [10].

Nevertheless, several important knowledge gaps persist. For example, it remains unknown whether conventional echocardiographic markers such as presence of mitral valve pathology, LA size, LV ejection fraction (LVEF), LV diastolic function or E/e′ can identify patients at higher risk of developing clinically meaningful MR after isolated tricuspid valve surgery. Given the paucity of comprehensive evidence, we conducted a single-center retrospective study in a well-defined Iranian cohort to characterize MR evolution following isolated TV surgery and to explore echocardiographic predictors of postoperative MR progression.

## Materials and methods

### Study design and population

This retrospective observational cohort was conducted at Rajaie Cardiovascular Medical and Research Institute (Tehran, Iran). All consecutive patients aged ≥ 14 years who underwent isolated tricuspid valve surgery, either repair or replacement for severe chronic tricuspid regurgitation (TR) or surgery for severe native tricuspid stenosis (TS), between February 2012 and December 2023 were screened for eligibility. In patients with traumatic tricuspid regurgitation, the condition was chronic at the time of surgery; none of the patients underwent intervention during the acute post‑traumatic phase.

### Inclusion criteria

Patients were eligible if they met all of the following:

- Underwent isolated surgical repair or replacement of the native tricuspid valve.
- Had echocardiographically confirmed severe chronic TR or severe native TS before surgery, documented according to ASE criteria.

### Exclusion criteria

Patients were excluded if any of the following were present preoperatively or immediately after surgery:

- LVEF ≤ 35%.
- Moderate or greater mitral regurgitation, or any mitral stenosis.
- Moderate or greater aortic regurgitation (AR) or aortic stenosis (AS).
- Systolic pulmonary artery pressure (SPAP) ≥ 50 mmHg.
- Moderate–severe postoperative right ventricular systolic dysfunction.
- Residual moderate or greater TR following tricuspid repair.
- Moderate or greater paravalvular leak after tricuspid valve replacement.
- Significant TS after surgery.
- Any concomitant cardiac procedure other than isolated tricuspid valve repair/replacement.
- Acute TR due to infective endocarditis or mass-related obstruction.
- Presence of a cardiovascular implantable electronic device.
- Prior mitral valve replacement.
- Percutaneous coronary intervention within 3 months before tricuspid surgery.

These criteria ensured a homogeneous cohort undergoing truly isolated intervention on the tricuspid valve.

### Clinical and echocardiographic data collection

Baseline demographic and clinical characteristics, including age, sex, body mass index, diabetes mellitus, hypertension, hyperlipidemia, smoking status, and coronary artery disease, were extracted from electronic medical records.

Transthoracic echocardiography (TTE) was performed by fellowship of echocardiography and interpreted according to ASE recommendations [12]. All preoperative and postoperative TTEs were independently reviewed by a single board-certified echocardiography expert blinded to all clinical outcomes.

Severity of mitral regurgitation (MR) and other valvular lesions was assessed according to ASE recommendations. In this retrospective study, MR severity was primarily determined using an integrated qualitative and semi‑quantitative approach based on available echocardiographic images and formal report data. Quantitative MR parameters (e.g., regurgitant volume, effective regurgitant orifice area, vena contracta width) were not consistently available for all patients and therefore were not systematically analyzed. MR grades were treated as ordinal categories based on the integrated qualitative severity classification. Because this was a retrospective study, original echocardiographic DICOM images were not consistently available for comprehensive morphologic analysis. Therefore, detailed mitral valve geometric parameters, including mitral annular diameter, leaflet angles, coaptation height, and tenting height, could not be systematically measured. MR severity was assessed using an integrated qualitative/semi-quantitative approach based on available images and formal reports.

### Preoperative echocardiographic assessment

The last preoperative TTE prior to surgery was analyzed. The following parameters were systematically recorded:

- LVEF and LV end-diastolic volume index (LVEDVi).
- LV internal dimensions.
- Presence and grade of diastolic dysfunction (ASE 2016).
- E/e′ ratio in mitral inflow.
- Left atrial (LA) size, LA area, and LA volume index (LAVI).
- RV size (quantitative and qualitative), RV systolic function, TR and TS severity.
- Etiology of TR or TS.
- Presence and severity of MR, AR/AS, and other valvular abnormalities.
- Presence of structural mitral leaflet pathology.
- SPAP and presence/severity of pulmonary hypertension.

### Operative details

Operative records were reviewed for:

- Procedure type (repair vs. replacement).
- In replacement cases, prosthesis type and size.

### Postoperative echocardiographic assessment

The most recent postoperative TTE performed within 12 months following surgery at the same center was used. Parameters recorded mirrored the preoperative dataset and additionally included:

- LVEF.
- Severity of MR.
- Residual or recurrent TR or TS severity.
- Degree of paravalvular leak (in case of prosthetic valve).
- Presence of significant prosthetic stenosis.
- Time interval (months) between surgery and the postoperative imaging study.

### Endpoints

The primary endpoint was worsening of MR, defined as an increase of ≥ 1 grade in MR severity between preoperative and postoperative TTE.

Secondary analyses investigated associations between MR progression and baseline characteristics in pre operation echocardiography including LVEF, LA size, LV diastolic dysfunction, E/e′, SPAP, presence of mitral leaflet pathology, and etiology of TR/TS.

### Statistical analysis

Statistical analyses were performed using SPSS v26.0 (IBM Corp., Armonk, NY).

- Continuous variables were expressed as mean ± standard deviation or median (IQR), based on Shapiro–Wilk normality testing.
- Group comparisons were conducted using Student’s t-test for normally distributed variables and Mann–Whitney U test for non-normally distributed data.
- Categorical variables were reported as frequencies (%) and compared using chi-square or Fisher’s exact test.
- Univariable logistic regression was performed to evaluate associations between individual preoperative parameters and MR worsening. Given the limited sample size and number of outcome events, multivariable logistic regression was not pursued in order to avoid model overfitting, in accordance with current recommendations for small datasets.
- All statistical tests were two-sided, with *P* < 0.05 considered significant.

### Ethical considerations

The study protocol was approved by the Ethics Committee of Rajaie Cardiovascular Medical and Research Center (IR.RHC.REC.1404.073). The requirement for informed consent was waived due to the retrospective nature of the study and use of anonymized clinical data.

## Result

A total of 19 patients who met all predefined criteria were included in the final analysis. All patients had undergone isolated surgical tricuspid valve repair due to severe chronic tricuspid regurgitation and no patient with TS had fulfilled inclusion criteria. All had complete paired preoperative and postoperative echocardiographic studies interpreted by a blinded expert reader. The mean age was 36.3 ± 14.0 years (range 17–67), and 63% were male. The median follow-up interval was 8.1 ± 7.7 months (range 1–24 months). Baseline demographic and echocardiographic characteristics are shown in Table 1.

**Table 1:** Summary of baseline variables for all enrolled patients, highlighting key demographic features and preoperative right- and left-heart measurements prior to tricuspid valve repair

Variable	Value
Age (years)	36.3 ± 14.0
Male (%)	84.2% (16/19)
Height (cm)	173.8 ± 7.8 (*n* = 11)
Weight (kg)	76.8 ± 9.6 (*n* = 11)
Body Surface Area (m²)	1.90 ± 0.11 (*n* = 13)
**Etiology of TR**	
Congenital/Ebstein/Cleft/Dysplastic	57.9%
Traumatic	21.1%
Degenerative/annular calcification/Prolapse	21.0%
**Pre-operative Echocardiographic Characteristics**	
Preop LVEF (%)	48.4 ± 5.3
Preop LVEDVi (mL/m²)	50.9 ± 10.7
Preop E/e′	7.62 ± 2.50
Preop LA area (cm²)	15.0 ± 3.29
Preop LAVI (mL/m²)	24.9 ± 7.98
Preop RV enlargement (qualitative)	57.9% (11/19)
Preop RVIDd(cm)	4.60 ± 0.58
Preop diastolic dysfunction (≥ 1 garde)	47.4% (9/19)

### Right-sided hemodynamics and RV remodeling

Preoperative systolic pulmonary artery pressure (SPAP) averaged 32.5 ± 5.5 mmHg, confirming the absence of significant pulmonary hypertension at study entry. Postoperative SPAP remained unchanged (32.2 ± 5.0 mmHg; *p* = 0.731).

In contrast, right-sided chamber dimensions demonstrated substantial remodeling. RV size qualitatively decreased in a statistically significant manner (*p* = 0.001). Quantitatively, right ventricular internal diameter at end diastole (RVIDd) declined from 4.60 ± 0.58 cm to 3.89 ± 0.44 cm (*p* < 0.001), representing the most pronounced structural improvement observed in the cohort.

Qualitative RV systolic dysfunction showed a nonsignificant trend toward improvement (*p* = 0.070). Together, these parameters reflect notable reverse remodeling of the RV after tricuspid valve repair.

### Tricuspid valve function

The severity of TR improved significantly after surgery (*p* < 0.001), with the majority of patients transitioning from severe preoperative regurgitation to mild or moderate residual TR.

Other cardiac valve parameters, including aortic regurgitation (AR; *p* = 0.317), aortic stenosis (AS; *p* = 1.000) did not meaningfully change postoperatively.

### Left-sided cardiac chambers structure and function

Left ventricular systolic function remained preserved throughout the study period, with no significant change in LVEF (48.4 ± 5.2% vs. 48.7 ± 4.4%; *p* = 0.796). LV chamber dimensions also remained stable, including LVIDd (4.54 ± 0.48 cm vs. 4.69 ± 0.51 cm; *p* = 0.149) and LVEDVi (*p* = 0.461).

Diastolic parameters showed no significant change overall. Average E/e′ increased minimally (7.62 ± 2.50 to 8.08 ± 1.91; *p* = 0.234), and categorical diastolic dysfunction grades were similar pre- and post-repair (*p* = 0.655).

Left atrial structure likewise remained stable: LA diameter (*p* = 0.567), LA area (*p* = 0.151), and LAVI (*p* = 0.401) showed no significant postoperative change. These findings confirm that left-sided chamber remodeling was limited overall.

### Mitral valve function and primary outcome (MR Progression)

Mitral regurgitation severity demonstrated a significant overall change in paired analysis (*p* = 0.010). When applying the predefined study definition of MR worsening (≥ 1-grade increase), 12 patients (63%) experienced postoperative MR worsening, while 7 patients (37%) demonstrated stability or improvement. A one-grade increase was defined as a change of one severity category on the predefined MR grading scale (e.g., from mild to mild-to-moderate).

Preoperative MR severity was absent in 21.1% of patients, mild in 63.2%, and mild-to-moderate in 15.8%. Postoperative assessment showed a shift toward higher MR grades, with mild MR in 47.4%, mild-to-moderate MR in 36.8%, and moderate MR in 15.8%. Overall, four patients (15.8%) demonstrated progression to ≥ moderate MR, all of whom fell within the moderate category; no cases of severe MR were observed. These data provide a clearer depiction of MR evolution within the cohort.Most deteriorations were modest (+ 1 grade), although a minority progressed by two or more grades. This high rate of postoperative MR change occurred despite preserved LV systolic function in paired analysis, and significant improvement in right-sided loading conditions.

### Group comparison: MR worsened vs. MR stable/improved

#### Demographics and clinical features

No significant differences were observed between groups with respect to age, sex, body size, diabetes, hypertension, smoking, coronary artery disease, or preoperative SPAP. Age showed a mild numerical difference (35.7 vs. 37.4 years) but was not statistically meaningful.

### Right-sided measures

Both groups exhibited universal preoperative RV dilation; however, severe preoperative RV enlargement was more common among patients with MR worsening (8 of 12 vs. 3 of 7). Postoperatively, moderate to severe dilation also remained more frequent in this group (10 of 12 vs. 4 of 7).

Despite these patterns, neither qualitative RV dysfunction nor quantitative RVIDd differences reached statistical significance in between-group testing.

Additional baseline indices of right ventricular systolic performance were also examined. Preoperative TAPSE was numerically higher in patients who developed MR worsening compared with those without worsening (24.0 ± 4.6 mm vs. 21.6 ± 2.6 mm), although the difference was not statistically significant (Mann–Whitney U = 32, *p* = 0.394).

### Left-sided filling pressures and chamber size

- Quantitative analysis revealed several clinically meaningful patterns:
- Preoperative E/e′: higher in patients with MR worsening (trend; *p* = 0.071).
- Postoperative E/e′: significantly higher in the MR-worsened group (9.39 ± 2.32 vs. 7.16 ± 0.65; *p* = 0.045).
- Postoperative LAVI: numerically larger among patients with MR worsening (34.1 vs. 22.3 mL/m²), although not statistically significant.
- LV volumes and diameters: showed no between-group differences.

These findings indicate that patients with postoperative MR progression exhibit a persistently different filling-pressure profile, even though the overall cohort showed virtually no change in paired E/e′ (mean 7.4 pre-operatively vs. 7.1 post-operatively).

Preoperative LVOT velocity–time integral (LVOT VTI), used as an index of left ventricular forward stroke volume, also did not differ between patients with and without MR worsening (18.3 ± 2.2 cm vs. 19.5 ± 3.1 cm; Mann–Whitney U = 34, *p* = 0.496). **Mitral and Tricuspid Valve Morphology**.

Baseline MV morphology (normal, prolapse, thick leaflet, degenerative) did not differ between groups. As per protocol, none of the patients had more than mild MR preoperatively. Residual or recurrent TR and TS severity also showed no group differences.

### Predictors of MR worsening

Univariable logistic regression was performed to explore potential preoperative predictors of postoperative MR worsening. None of the evaluated variables reached statistical significance. Preoperative LVEDVi (OR 1.11; 95% CI 0.97–1.27; *p* = 0.111), E/e′ (OR 1.81; 95% CI 0.72–4.56; *p* = 0.209), age (OR 0.96; *p* = 0.233), and preoperative SPAP (OR 1.065; *p* = 0.501) showed no significant associations. Similarly, preoperative RV size (OR 3.00; *p* = 0.198), LAVI (OR 1.131; *p* = 0.190), MV pathology (OR 0.923; *p* = 0.873), and RV dysfunction (OR 0.750; *p* = 0.640) were not associated with MR worsening.

Multivariable logistic regression was not performed due to the very small number of events, as such a model would be statistically unstable. Full univariable regression results are summarized in Tables 3 and 4.

We examined the association between preoperative diastolic dysfunction and postoperative MR progression. MR worsening occurred in 7 out of 9 patients (77.8%) with preoperative diastolic dysfunction, compared with 5 out of 10 patients (50.0%) without diastolic dysfunction. This difference was not statistically significant (Fisher’s exact *p* = 0.35).

## Discussion

Isolated tricuspid regurgitation (TR) has increasingly been recognized as a clinically meaningful condition, with population-level prevalence estimates reported between 5% and 20%, and with severity strongly linked to morbidity and mortality [3, 13]. Historically, management for symptomatic TR largely relied on diuretics and volume control, yet these approaches have shown no demonstrable mortality benefit [3, 14]. Surgical correction, although physiologically intuitive, has long been approached with caution due to earlier reports describing substantial perioperative mortality and morbidity, especially when compared with left-sided valve surgery [6, 8, 14]. Over time, however, more contemporary analyses have shown that isolated tricuspid operations now represent a growing proportion of valve surgeries, with in-hospital mortality in the 7–10% range and consistent with international registries [15, 16]. Collectively, these data highlight both the clinical relevance of isolated TR and the need to understand postoperative valvular interactions, particularly mitral regurgitation (MR) progression, which may have significant downstream implications.

Within this framework, our study evaluated 19 patients undergoing isolated tricuspid valve repair with complete pre- and postoperative echocardiograms reviewed by a blinded expert reader. The cohort was younger than typically observed in national registries [2, 10], with a mean age of 36 years and a predominance of congenital and traumatic etiologies, as shown in Table 1. This distinguishes our population from administrative cohorts that generally consist of older individuals with higher comorbidity burdens [2, 8, 16]. All included patients met strict criteria, most notably LVEF ≥ 40% and absence of significant pulmonary hypertension.

### Primary findings: MR worsening is frequent and statistically significant

A central finding of our study is that 63.2% of patients experienced worsening MR after isolated tricuspid repair, using the study’s predefined endpoint of ≥ 1 grade increase. Although the observed proportion is somewhathigher than the 9–31% range reported in earlier investigations of isolated TR intervention. One study reviewing standalone tricuspid surgery found significant MR progression in 15.5% of patients within 3–12 months, attributing all cases to annular dilation [10]. The younger age of our cohort, narrower grading of MR and the predominance of congenital etiologies may partially explain discrepancy, but our data suggest that MR worsening may occur in a subset of patients following isolated TV repair.

The paired statistical analysis confirmed that MR grade changed significantly (*P* = 0.010). This stands in contrast with parameters such as LV size, LV systolic function, LVEDVi, and diastolic indices, none of which showed significant paired changes. This pattern may suggest that MR worsening is not simply a reflection of global LV deterioration but could also relate to more specific hemodynamic interactions.

### RV remodeling and hemodynamic shifts: significant improvements but complex effects on the mitral valve

One of the strongest physiologic signals in our data is the significant improvement in right-sided chamber structure following tricuspid repair. Both qualitative RV size (*P* = 0.001) and quantitative RVIDd (4.60 ± 0.58 cm to 3.89 ± 0.44 cm; *P* < 0.001) showed marked improvements, among the most statistically robust findings in this cohort (Table 2). TR severity also improved significantly (*P* < 0.001), reflecting successful surgical restoration of valvular competence.

**Table 2:** Wilcoxon signed ranks analysis comparing pre- and postoperative echocardiographic measurements, illustrating marked reductions in TR and RV size alongside stable LV structure and function

Parameter	Pre-op mean ± SD	Post-op mean ± SD	P-value
RV size (qualitative)	Dilated	Improved	0.001 *
RVIDd (cm)	4.60 ± 0.58	3.89 ± 0.44	< 0.001 *
TR severity	Severe	Improved	< 0.001 *
LVEF (%)	48.4 ± 5.3	48.7 ± 4.4	0.796
LVEDVi (mL/m²)	50.9 ± 10.7	52.3 ± 9.6	0.234
LVIDd (cm)	4.54 ± 0.48	4.69 ± 0.51	0.234
E/e′	7.62 ± 2.50	8.08 ± 1.91	0.234
LA diameter (cm)	3.20 ± 0.60	3.27 ± 0.60	0.514
LA area (cm²)	15.0 ± 3.29	17.8 ± 8.53	0.112
LAVI (mL/m²)	24.9 ± 7.98	29.1 ± 25.6	0.197
SPAP (mmHg)	32.5 ± 5.5	32.1 ± 5.0	0.731

* Statistical significance was defined as *p* < 0.05

These observations contrast markedly with prior large administrative reports noting that patients undergoing isolated tricuspid intervention typically present late, with longstanding volume overload and incomplete reversibility of RV remodeling [8, 17]. Although our population was younger and thus more likely to benefit from reverse remodeling, the postoperative increase in forward RV output may still exert physiological influence on the left heart. Indeed, previous detailed case descriptions have highlighted how increased RV output following TR correction results in elevated LV preload, LV dilation, and consequent mitral leaflet tethering [9, 18]. In one example, increases in left ventricular outflow tract (LVOT) and right ventricular outflow tract (RVOT) velocity-time integral (VTI)corroborated with the hemodynamic shift [9]. Our study did not measure LVOT VTI systematically, but the significant RV remodeling and TR reduction observed in Table 2 supports this mechanism conceptually. In our dataset, baseline TAPSE and LVOT VTI were available for all patients; however, neither index differed significantly between groups with and without MR worsening (both *p* > 0.39), suggesting that preoperative global RV systolic performance and LV forward flow were not primary determinants of postoperative MR behavior in this small cohort.

As mentioned before, in our study, MR progression after TV surgery was more prevalent than previous studies. This can be due to this significant improvement in RV function after surgery in our young study population in contrast with previous studies, that intensified RV output and left heart preload besides TR correction effect.

### Role of LV filling pressures and LA size: E/e′ as the only statistically significant between-group difference

Perhaps the most important new finding from the updated analysis is the significant postoperative difference in E/e′ between patients with MR worsening and those without (9.39 ± 2.32 vs. 7.16 ± 0.65, *P* = 0.045, Table 3). This is the only parameter with statistically significant between-group separation.

**Table 3:** Comparison of echocardiographic parameters according to MR Worsening

Parameter	MR Worsened (*n* = 12) – Pre-op	MR Stable (*n* = 7) – Pre-op	P (Pre-op)	MR Worsened (*n* = 12) – Post-op	MR Stable (*n* = 7) – Post-op	P (Post-op)
**E/e′**	8.7 ± 2.5	7.2 ± 0.7	0.154	9.39 ± 2.32	7.16 ± 0.65	0.045**
**LAVI (mL/m²)**	27.2 ± 10.5	21.5 ± 4.2	0.168	35.8 ± 37.2	21.4 ± 3.9	0.314
**RVIDd (cm)**	4.66 ± 0.69	4.72 ± 0.39	0.852	3.71 ± 0.39	4.17 ± 0.38	0.046*
**LVEDVi (mL/m²)**	55.4 ± 11.7	45.7 ± 6.9	0.063	54.1 ± 10.0	49.3 ± 10.7	0.404

* Statistical significance was defined as *p* < 0.05

Although paired analysis did not reveal a significant pre/post change in E/e′ across the full cohort (*P* = 0.234), the between-group divergence suggests differential susceptibility to loading conditions among individuals. This aligns with prior literature: elevations in left atrial (LA) pressure can reduce atrioventricular pressure gradients and weaken mitral closing forces, exacerbating MR [19, 20]. Earlier reports have also proposed that chronic diastolic abnormalities may potentiate MR progression after TR correction [19–21].

In line with these models, patients who developed worsening MR in our study also showed:

- higher preoperative E/e′ (trend, *P* = 0.071),
- higher postoperative E/e′ (significant, *P* = 0.045),
- numerically larger postoperative LAVI (34.1 vs. 22.3 mL/m²),
- numerically greater persistence of RV dilation.

Even though the latter parameters did not reach statistical significance, the directional alignment with established pathophysiology may indicate a potential association with LV filling pressures, although this observation should be interpreted cautiously.

These findings are consistent with a few previous observations. Studies evaluating MR progression after tricuspid surgery noted that larger LA volume index and impaired LA strain were predictors of postoperative MR deterioration [10, 21, 22]. While LA strain was not measured here, our LAVI trend and preoperative E/e′ trend difference is concordant with these insights.

**Table 4:** Univariable logistic regression for predictors of postoperative MR worsening

Variable	OR	CI 95%/SE	P-value
**Pre op LVEDVi**	1.11	0.97–1.27	0.111
**Pre op E/e`**	1.81	0.72–4.56	0.209
**Age**	0.96	0.89–1.03	0.233
**Pre op SPAP**	1.065	0.102	0.501
**Pre op RV size**	3.00	0.771	0.198
**Pre op LAVI**	1.131	0.088	0.190
**MV pathology**	0.923	1.145	0.873
**Pre op RV dysfunction**	0.750	0.646	0.640

Statistical significance was defined as *P* < 0.05

### Septal mechanics, Bi-ventricular interdependence, and valve geometry

The interplay between TR correction and MR has also been explored in transcatheter studies, where variations in septal geometry and ventricular interdependence appear to modulate MR outcomes [23–26]. Checkpoints from TTVI research underscore how chronic TR can elevate right-sided pressures, shift the interventricular septum to left side, and impair LV compliance. Successful TR reduction may normalize septal geometry, improving MR as demonstrated in the HERACLES HFpEF trial [23, 27]. Conversely, certain interventions such as annuloplasty or heterotopic valve replacement were associated with MR worsening, emphasizing the importance of geometric perturbation [23].

Even though our study involves surgical TV repair, these models help interpret our findings, particularly the trend toward larger preoperative RV size in the MR-worsening group (8 of 12 vs. 3 of 7) and significant improvement in RV size in both MR worsening group and no MR worsening. This could be explained by longstanding effect of RV enlargement on septal curvature and irreversible reduced LV compliance despite reduced RV size after surgery, resulting in MR progression.

However, due to the retrospective design and incomplete availability of raw DICOM images, we could not systematically quantify mitral valve geometry (annular size, leaflet angles, or tenting height), which should be addressed in future prospective studies.

### Mitral valve morphology: limited structural burden but not irrelevant

Degenerative mitral disease was uncommon and mild in our population, consistent with the young age distribution, and Table 1 shows no significant between-group differences in morphology. However, even minor leaflet abnormalities can become more clinically consequential under increased LV preload or altered papillary muscle geometry, playing a secondary role in susceptibility to MR worsening, as suggested by previous systematic evaluations [9, 28]. However, the patients were also stratified according to the presence of baseline mitral valve (MV) pathology based on qualitative echocardiographic assessment (normal vs. any structural leaflet abnormality such as prolapse, thickening, or degenerative MAC changes). MR worsening occurred in 8 of 13 patients (61.5%) without MV pathology and in 2 of 6 patients (33.3%) with MV pathology. In a 2 × 2 analysis, the presence of MV pathology was not significantly associated with postoperative MR worsening (odds ratio 0.31; 95% CI wide, *p* = 0.35, Fisher’s exact test). Notably, among patients who experienced MR worsening, mitral valve prolapses represented the most prevalent baseline leaflet abnormality, being present in 4 of 12 patients (33.3%). Thus, in this small cohort, baseline structural MV abnormalities did not emerge as a significant determinant of MR progression after isolated tricuspid valve repair. Our findings, therefore, remain physiologically compatible with these mechanisms despite the absence of statistical significance in morphological variables.

### Regression analysis: no independent predictors, but E/e′ emerges as a directional marker

Multivariable regression in this small cohort did not identify independent predictors of MR worsening in patients that underwent TV surgical repair. This is unsurprising given the limited event-to-variable ratio and the modest overall sample size. However, preoperative E/e′ produced the strongest directional association (OR = 3.53), reinforcing its biological plausibility as a surrogate for LV filling pressures and LA pressure. Although not statistically significant, its consistency with prior mechanistic pathways [9, 10, 23] supports its inclusion as a candidate parameter for risk stratification in future, larger studies. Notably, prior studies have identified several preoperative markers of MR progression after isolated TV interventions—including larger LA volume, impaired LA strain, and higher baseline E/e′ in surgical cohorts—as well as transcatheter-specific predictors such as annuloplasty-based TTVI and heterotopic valve replacement. Together, these findings suggest that indices of elevated filling pressures and atrial–annular remodeling represent the most reproducible predictors of postoperative MR worsening across both surgical and transcatheter datasets [10, 23].

### Clinical implications

Persistent or worsening MR after TR intervention has been associated with higher risk of adverse clinical outcomes, including heart failure hospitalization and atrial arrhythmia in larger series [23]. Given the high rate of MR worsening (63.2%) and its significant correlation with postoperative E/e′, close echocardiographic follow-up during the first postoperative year is warranted, particularly for patients with elevated filling pressures, larger atrial dimensions, or more advanced RV remodeling.

Prior reports have also proposed intraoperative loading tests to identify latent mitral insufficiency immediately after TR repair [9, 15]. Although such approaches remain understudied, the physiologic framework of our findings, especially the role of filling pressures, supports the rationale for considering more dynamic intraoperative assessments in selected high-risk patients.

### Summary

In summary, this study provides several important contributions:

1. MR worsening is common (63.2%) after isolated surgical tricuspid valve repair in a young, predominantly congenital population.
2. MR severity changes significantly in paired analysis (*P* = 0.010), despite stability in most LV indices.
3. RV remodeling is robust and statistically significant, with marked reductions in both RV size and RVIDd (*P* = 0.001 and *P* < 0.001).
4. Postoperative E/e′ is significantly higher among patients with MR worsening (*P* = 0.045), the only between-group variable reaching statistical significance, highlighting the role of LV filling pressures.
5. Trends in LAVI, RV dimensions, and preoperative E/e′ are physiologically coherent with mechanistic literature [9, 15, 23, 29], though not statistically significant
6. No single independent predictor emerged in multivariable analysis, underscoring the multifactorial nature of postoperative MR dynamics.

These findings emphasize the need for comprehensive preoperative assessment, careful evaluation of filling pressures, and vigilant postoperative echocardiographic monitoring. Larger, multicenter studies employing standardized imaging protocols, including LA strain, LVOT VTI, septal curvature analysis, and dynamic diastolic evaluations, are necessary to better delineate predictors of MR evolution and to optimize perioperative strategies aimed at mitigating postoperative mitral dysfunction.

### Limitations

This study has several limitations that should be considered when interpreting the findings. First, the sample size was small, reflecting the rarity of isolated tricuspid valve surgery in younger patients and the strict inclusion criteria employed. As a result, the statistical power to detect modest associations was limited, and several directional trends, such as preoperative E/e′, postoperative LAVI, and the distribution of RV enlargement, did not reach statistical significance despite physiologic plausibility. Second, the retrospective design introduces the possibility of selection bias, particularly regarding referral patterns and the timing of postoperative echocardiography, which varied across patients. Although all studies were interpreted by a single blinded expert reader, interobserver variability could not be assessed.

Third, echocardiographic parameters such as LVOT VTI, RV functional indices (TAPSE, FAC, S′), detailed septal curvature analysis, and left atrial strain were not uniformly available, limiting detailed mechanistic assessment, particularly regarding ventricular interdependence and LA function. The absence of these variables restricts the ability to infer mechanistic pathways and may have contributed to the inability of multivariable regression to identify independent predictors of MR worsening. Fourth, the classification of diastolic dysfunction followed standard Doppler indices but lacked invasive validation, which may reduce the precision of filling pressure estimation. Fifth, although our cohort was homogeneous in terms of preserved LV systolic function and absence of pulmonary hypertension, this also limits generalizability to older or comorbidity-burdened populations typically represented in national registries.

Finally, the study did not include long-term clinical outcomes, such as heart failure hospitalization, arrhythmic events, or reintervention, preventing assessment of the prognostic impact of postoperative MR progression. Future prospective, multicenter studies with standardized imaging protocols, larger sample sizes, and longitudinal follow-up are needed to validate these findings and to refine predictors of MR evolution after isolated tricuspid valve repair.

## Conclusion

In this small cohort of young patients undergoing isolated tricuspid valve repair, postoperative mitral regurgitation showed mild progression in a subset of individuals. While right-sided reverse remodeling was consistently observed, left-sided chamber size and systolic function remained largely stable. Patients with MR progression demonstrated higher postoperative E/e′ values, although this association should be interpreted cautiously and viewed as exploratory given the limited sample size and lack of multivariable modeling. These findings generate hypotheses regarding the influence of loading conditions on postoperative MR behavior and highlight the need for larger studies to better define clinical relevance and underlying mechanisms.

## Data Availability

All data supporting the findings of this study are included within this article. Additional datasets generated and/or analyzed during the current study are available from the corresponding author upon reasonable request.

## Abbreviations

AR: Aortic regurgitation
AS: Aortic stenosis
ASE: American society of echocardiography
BSA: Body surface area
CAD: Coronary artery disease
E/e′: Ratio of early mitral inflow velocity to mitral annular early diastolic velocity
HFpEF: Heart failure with preserved ejection fraction
IQR: Interquartile range
iTR: Isolated tricuspid regurgitation
LA: Left atrium
LAVI: Left atrial volume index
LV: Left ventricle
LVEDVi: Left ventricular end–diastolic volume index
LVEF: Left ventricular ejection fraction
LVIDd: Left ventricular internal diameter at end–diastole
MAC: Mitral annular calcification
MR: Mitral regurgitation
MV: Mitral valve
PISA: Proximal isovelocity surface area
RV: Right ventricle
RVIDd: Right ventricular internal diameter at end–diastole
SPAP: Systolic pulmonary artery pressure
TR: Tricuspid regurgitation
TS: Tricuspid stenosis
TTE: Transthoracic echocardiography
TV: Tricuspid valve
TVSx: Tricuspid valve surgery
TTVI: Transcatheter tricuspid valve intervention
VTI: Velocity–time integral

## Declarations

### Clinical trial number

Not applicable. This study is a retrospective observational analysis and was not registered as a clinical trial.
